# Correlation of circulating miRNA-33a and miRNA-122 with lipid metabolism among Egyptian patients with metabolic syndrome

**DOI:** 10.1186/s43141-021-00246-8

**Published:** 2021-10-05

**Authors:** Miral M. Refeat, Naglaa Abu-Mandil Hassan, Inass Hassan Ahmad, Eman Roshdy Mohamed Mostafa, Khalda S. Amr

**Affiliations:** 1 Medical Molecular Genetics Department, Human Genetics and Genome Research Division, National Research Centre, Cairo, Egypt; 2 Bilogical Anthropology Department, Medical Division, National Research Centre, Cairo, Egypt; 3 Endocrinology Department, Medicine for Girls Faculty, Al-Azhar University, Cairo, Egypt; 4 Internal Medicine Department, Medicine for Girls Faculty, Al-Azhar University, Cairo, Egypt

**Keywords:** Metabolic syndrome, MicroRNA, miR-33a, miR-122, Type 2 diabetes, Obesity

## Abstract

**Background:**

Metabolic syndrome is defined as a group of interrelated biochemical, clinical, and metabolic factors that directly increase the risk of cardiovascular disease, obesity, and type 2 diabetes mellitus. MicroRNA-33a (miR-33a) and MicroRNA-122 (miR-122) play a crucial role in various biological processes by regulating the gene expression level through post-transcriptional mechanisms, and alterations of their levels are associated with lipid and glucose metabolic disorders. In the present study, we aimed to investigate the correlation of miR-33a and miR-122 with obesity indices and glycemic parameters in a cohort of Egyptian patients. Quantitative real-time polymerase chain reaction (RT-PCR) using TaqMan assay was carried out to estimate the expression levels of miR-33a and miR-122 in serum samples of 100 patients diagnosed as having metabolic syndrome and 50 healthy controls. All patients (100%) had type 2 diabetes (by both history and laboratory assessment) and 70% were obese (*BMI* ≥ 30 kg/m^2^).

**Results:**

Compared to controls, patients had significantly higher serum expression level of miR-33a (*p* value < 0.001) and miR-122 (*p* value = 0.0016). miR-33a was less expressed (downregulation expression) with 0.8 fold change in the patient group (obese and diabetic) compared to healthy controls, while miR-122 was highly expressed (upregulation expression) in the patient group of patients with 1.9 fold change. Clinical parameters as body mass index (*BMI*), wrist circumference (Wc), weight (Wt), and height (Ht) (all *p* < 0.001); total cholesterol (TC) (*p* = 0.0115); and triglyceride (TG) (*p* = 0.0286), all were significantly higher in patients compared to the healthy group. Both miRNAs show statistically significant correlations with clinical and biochemical parameters (*p* < 0.001).

**Conclusions:**

Circulating miR-33a and miR-122 might be convincing as possible biomarkers for the diagnosis of metabolic syndrome.

## Background

Metabolic syndrome (MetS) is a cluster of metabolic disorders that includes hypertension, central obesity, cardiovascular disease, and diabetes mellitus [1]. The worldwide incidence of MetS ranges from 10 to 84% of the population, depending on the region, environment, sex, and age [2]. In Egypt, MetS reaches about 7.4% among the Egyptian population and it affects about 1 in 4 people in the Middle East [3]. Insulin resistance, visceral adiposity, dyslipidemia, genetic susceptibility, elevated blood pressure, and chronic stress represent the main factors which correlate to metabolic disorders. Glucose and lipid abnormalities are the two most common metabolic derangements of metabolic syndrome. Understanding pathway mechanisms involved in glucose and lipid metabolism would open new modalities in the diagnosis and management of metabolic syndrome [4]. microRNAs are small single-stranded non-coding RNA molecules that comprise 20–25 nucleotides with a transcriptional and posttranscriptional regulatory role in gene expression. They are involved in several processes, including lipid metabolism and insulin sensitivity. Studies have found that the expression level of the miRNA reflects its role associated with different disorders. Alterations of miRNA expression levels contributed to various diseases, such as obesity and diabetes mellitus [5]. Importantly, a single miRNA can regulate the expression of hundreds of genes and the expression of a single gene can be regulated by multiple miRNAs. So, miRNAs might act as ideal biological markers for rapid diagnosis, prognosis, and therapeutic mediators in metabolic disorders [6]. Specific miRNAs, including miRNA-33 and miRNA-122, were determined to have important roles in the regulation of lipid and glucose metabolism pathways [7]. miR-33 family is an intronic miRNA encoded by Srebp genes, located in intron-16 within two protein-coding genes for Sterol regulatory element-binding proteins (*SREBF*), *SREBP-2* and *SREBP-1* respectively, and it consists of two members named miR-33a and miR-33b [8]. miR-33 plays an important role in the regulation of cholesterol efflux, fatty acid metabolism, and insulin signaling. In concert with their host genes, *Srebp2* and *Srebp1*, miR-33a and miR-33b act to increase intracellular cholesterol and fatty acid levels by balancing transcriptional induction and posttranscriptional repression of lipid metabolism genes [9]. However, miR-33a and miR-33b affect glucose metabolism through pyruvate carboxy kinase (PCK1) and glucose-6-phosphate (G6PC) pathways, and they also control the expression of sirtuin 6 (Sirt6) and insulin receptor substrate 2 (IRS-2) and therefore regulate blood glucose levels [10]. Approximately 75% of total liver miRNA expression belongs to miRNA-122, the most abundant miRNAs in the liver. miR-122 plays an essential role in the maintenance of liver function through gene expression regulation, causing reduction of total cholesterol levels, HDL, apolipoprotein, LDL, and apolipoprotein B [11] by affecting regulatory enzymes involved in cholesterol biosynthesis. In glucose pathways, miR-122 reduces lactate production and increases oxygen consumption, by targeting many of glycolytic genes, especially pyruvate kinase (PK) gene [12].

As the two miRNAs are widely involved in lipid and glucose metabolism, they could be used as the biomarkers for the diagnosis of glucose and lipid metabolic disorders [13]. Although there is still a long way before we can use miR-33 and 122 effectively in clinical pathology, the application of these two miRNAs in the biomedical treatment of glucose and lipid metabolic disorders is significant and promising in the future [12]. The aim of this study is to investigate the associations of genetic expression of miR-33a and miR-122 in glucose and lipid metabolism as well as their correlations with metabolic syndrome parameters including obesity and type 2 diabetes (T2DM) in a cohort of Egyptian patients.

## Methods

### Ethics statement

The research protocol was approved by the ethical review committee of the Faculty of Medicine for Girls, Al-Azhar University institutional review board, Cairo, Egypt (AFMG IRB), reference number: 202001093. Sharing was voluntary; an informed written agreement was obtained from each participant before enrollment into the study. Data were anonymous and coded to assure the confidentiality of participants.

### Participants

This study enrolled 100 Egyptian patients selected by random sampling technique (males and females), diagnosed as having metabolic syndrome based on the National Cholesterol Education Programmed Adult Treatment Panel III (NCEP ATP III) [14]. They were selected from the outpatient clinic of Endocrinology at Al-Zahraa University Hospital, in Cairo, Egypt. Their age ranges were 34–60 years with a mean average of 48.45 ± 8.06. Fifty apparently healthy volunteers, recruited from paramedical personnel, served as controls of age ranges from 38 to 59 years (47.4 ± 4.2046). Patients and healthy volunteers were subjected to detailed medical and family history. Anthropometric measurements including height, weight, and *BMI* [weight (kg)/height (m^2^)] were done using standard protocols [15].

### Blood sample collection

Peripheral blood (6 mL) was collected from each participant as follows: 2 mL blood was taken into NaF tubes for blood glucose estimation, 2 mL for HbA1c test, and 2 mL for serum miRNAs. Four milliliters was taken independently into non-gel serum tubes for measuring lipid profile.

### Laboratory analysis

For lipid profile, blood was centrifuged at 3000×g for 15 min. Glycosylated Hb (HbA1c) was evaluated by quantitative colorimetry (Stanbio Laboratory, Boerne, TX, USA). Fasting blood glucose (FBG), total cholesterol (TC), high-density lipoprotein (HDL), and triglycerides (TG) were estimated by standard techniques (Olympus automatic analyzer AU 2700, Irish Branch, Ireland) and low-density lipoprotein (LDL) was determined by Friedewald formula (LDL = TC − TG/5 − HDL) [16].

### miRNA extraction

Serum was extracted by centrifugation at 2000 rpm for 15 min at 8 °C, and the supernatant was transferred into new tubes and stored at −80 °C till further proceeding. miR-33a and miR-122 were isolated from the serum using miRNeasy kit (Qiagen, USA) according to the manufacturer’s instructions. The concentration of the extracted miRNA had been quantified using NanoDrop and stored in aliquots at − 20 °C. miR-39 (Qiagen, USA) was used to normalize the expression levels of target miRNAs.

### Quantitative reverse transcription

For miRNA-specific reverse transcription, quantitative real-time polymerase chain reaction (qRT-PCR) assays of miR-33a and miR-122 were performed using TaqMan® MicroRNA kit (Lot: 4453320, Applied Biosystems, USA) according to manufacturer’s protocol and reactions were proceeded in step one real-time PCR system (Applied Biosystems, USA). The reaction was performed in a total volume of 25 μL and contained 100 ng of cDNA template, 1X of 20✕ TaqMan® Gene Expression Assay, 1X of 2✕ TaqMan® Gene Expression Master Mix, and complete volume up to 25μL with RNase-free water. Amplification program: 94 °C for 10 min followed by 40 cycles of 94 °C for 20 s then 60 °C for 30 s. Relative quantification (Rq) of miRNAs’ expression was calculated using the 2−ΔΔCT method as 2 − (mean patient ∆Ct − mean control ∆Ct). ΔCt was verified by subtracting the Ct (threshold cycle) values for endogenous control miR-39 from the Ct values for the gene of interest [17].

### Statistical analysis

Data were statistically analyzed using SPSS version 22.0 software (SPSS Inc., Chicago, IL, USA). A *p* value of less than 0.05 was considered statistically significant. Student *T* test was used to compare gene expression levels between groups, and correlations between gene expression levels and clinical parameters were analyzed using the Spearman rank correlation coefficient. Clinical data was presented as the mean ± standard deviation (*SD*) [18].

## Results

### Clinical and biochemical analysis

**Fig. 1:**
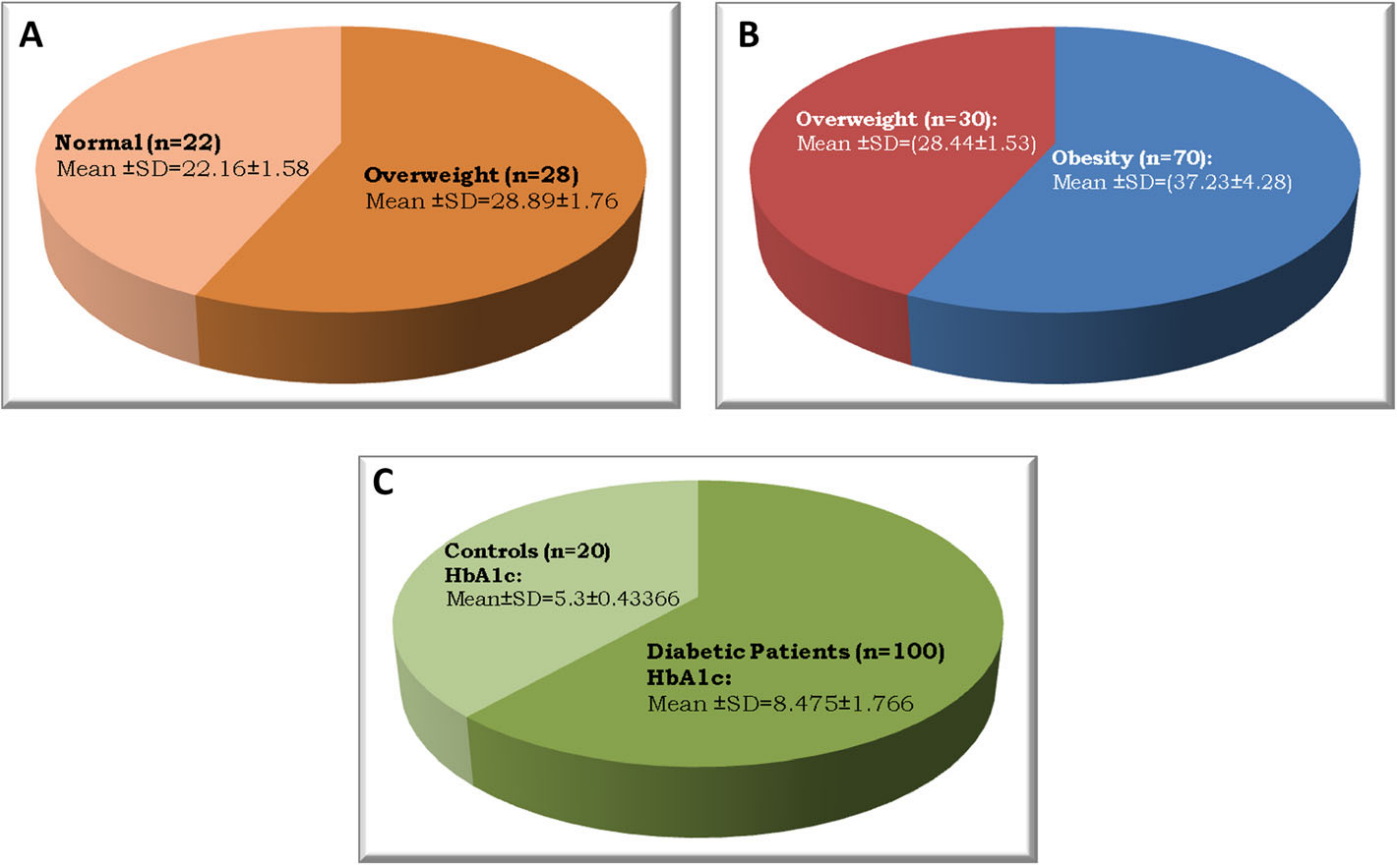
Schematic representation of the distribution of controls and diabetic patients with BMI and HbA1. **A** Body mass index in healthy controls divided to normal (*n* = 22, 44%) and overweight (*n* = 28, 56%) and **B** body mass index in patients divided to overweight (*n* = 30, 30%) and obese (*n* = 70, 70%) individuals. **C** Contribution of glycosylated Hb (HbA1c) in controls (*n* = 50) and diabetic patients (*n* = 100)

This study comprised 100 Egyptian patients, and their age ranged from 34 to 60 years and 50 healthy control of age ranged from 38 to 59 years. HDL shows significantly lower values in patients compared to controls with *p* values = 0.0394. However, all other parameters showed significantly higher values in patients compared to controls with *p* value ranges from < 0.001 to 0.0469. Seventy-five percent of patients were females and 25% were males while in control subjects 85% were females and 15% were males. According to body mass index (*BMI*), 30% of patients were overweight (*BMI* = ≥25–< 30 kg/m^2^) and 70% were obese (*BMI* = ≥30 kg/m^2^) while in control subjects 44% were of normal weight (*BMI* = ≥18–< 25 kg/m^2^) and 56% were overweight. All patients were diabetics with mean glycosylated Hb (HbA1c) 8.47575 ± 1.766534 (Table 1 and Fig. 1).

**Table 1:** Anthropometric measurements and laboratory findings among the study participants

Characteristics	Controls (***n*** = 50)	Patients (***n*** = 100)	***p***-value (< 0.05) is significant	Significance
**miRNA-33a**	0.0874 ± 0.08755	0.0037 ± 0.009579	< 0.001	HS
**miRNA- 122**	1.9615 ± 2.62465	3.938774 ± 3.938774	0.0016	HS
**Age (years)**	47.4 ± 4.2046	48.4571 ± 8.06350	< 0.001	HS
**Sex (male/female)**	**F** 75% **M** 25%	**F** 85% **M** 15%	– –	
**BMI (kg/m**^**2**^**)** **Normal weight ≥18–< 25** **Overweight ≥25–< 30** **Obese ≥30**	24.865 ± 6.1954 Normal (no = 22) (44%) Overweight (no = 28) (56%)	34.57 ± 5.01458 Overweight (*n* = 30) (30%) Obese (*n* = 70) (70%)	< 0.001	HS
**FBG (mg/dL)**	114.8 ± 47.02168	189.878 ± 66.9303	0.0140	HS
**HbA1c (%)**	5.3 ± 0.433669	8.47575 ± 1.766534	0.0469	HS
**TC (mg/dL)**	172.15 ± 50.2103	203.7273 ± 44.2141	=0.0115	HS
**TG (mg/dL)**	133.8 ± 58.01869	178.3636 ± 74.463	=0.0286	HS
**cHDL (mg/dL)**	42.5697 ± 9.78827	40.4 ± 8.987711	=0.0394	HS
**cLDL (mg/dL)**	100.86 ± 42.86601	125.803 ± 38.72177	=0.0211	HS

*BMI* body mass index, *FBG* fasting blood glucose, *HbAlc* glycosylated hemoglobin, *TC* total cholesterol, *TG* triglycerides, *cHDL* high-density lipoprotein cholesterol, *cLDL* low-density lipoprotein cholesterol, *F* female, *M* male, *HS* high significance. Data presented as mean ± SD value and *p* value

### Expression pattern of miRNA-33a in patients

The expression level of miR-33a was significantly higher with a *p* value < 0.001 in patients compared to the control group. miR-33a showed lower expression (downregulation expression) in patients compared to controls with 0.8 fold change.

### Expression pattern of miRNA-122 in patients

The serum expression level of miR-122 was significantly higher with a *p* value = 0.0368 in patients compared to the control group. miR-122 showed higher expression (upregulation expression) in patients compared to controls with 1.9 fold change.

### Expression of miRNA-33a and miRNA-122 in correlation with biochemical criteria

**Fig. 2:**
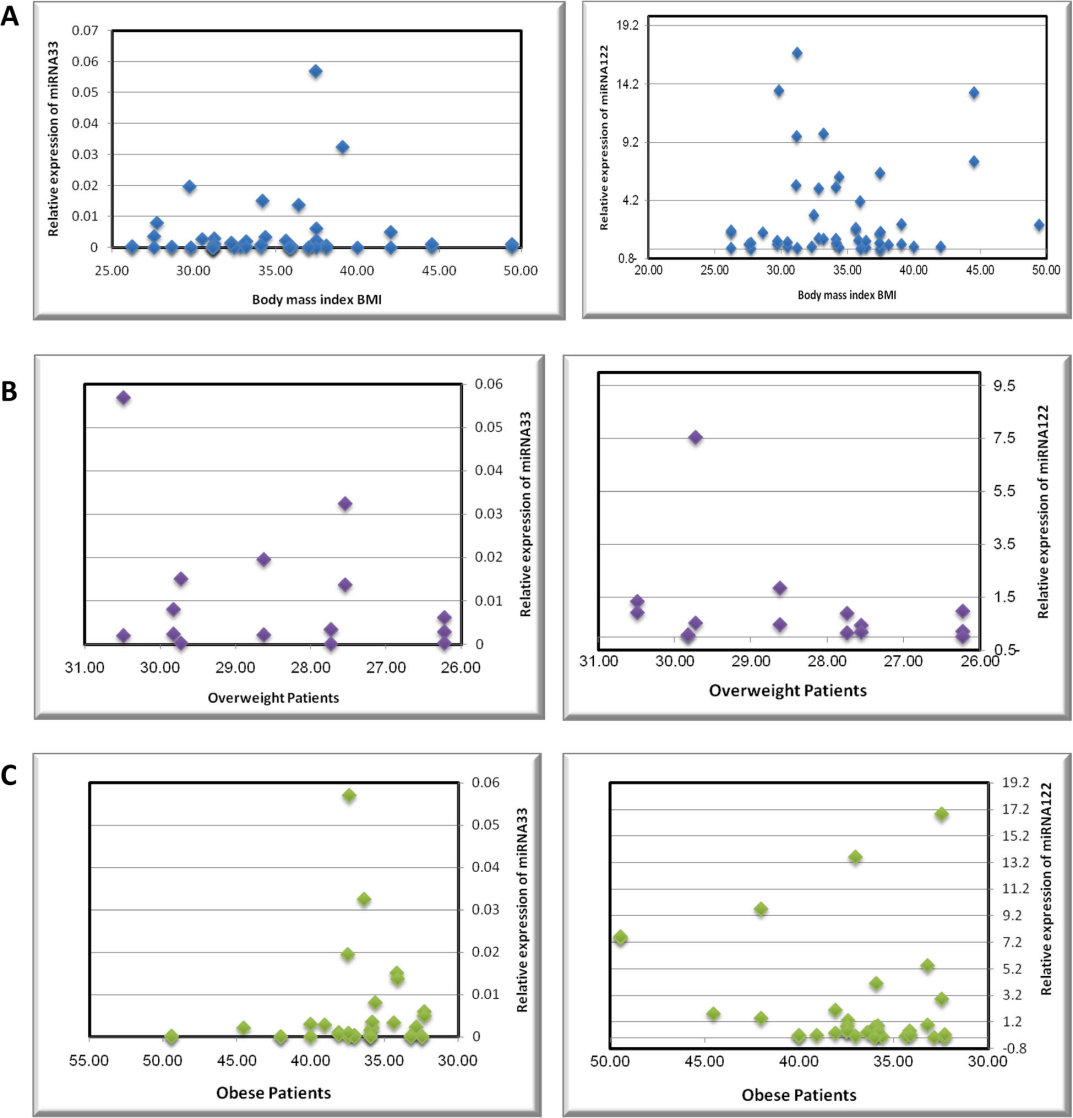
Correlation of relative expression of miRNA-33 and miRNA-122 with body mass index (**A**), including overweight (*n* = 30) (**B**) and obese patients (*n* = 70) (**C**)

Spearman rank correlation (*R*s) of miR-33a and miR-122 with biochemical parameters in patients revealed positive correlations of both miRNAs with glycosylated Hb (HbA1c) (*r* = 0.885 and 0.965) and fasting blood glucose (FBG) (*r* = 0.731 and 0.863 of *p* < 0.001). Meanwhile, there were moderate positive correlations between both miRNAs and lipid profile of patients including triglycerides (TG) (*r* = 0.342 and 0.291 and *p* < 0.001), high-density lipoprotein (HDL) (*r* = 0.149 and 0.268 and *p* < 0.001), and low-density lipoprotein (LDL) (*r* = 0.115 and 0.298 and *p* < 0.001). Correlations of miRNA-33a and miR-122 with BMI were positively highly significant (*r* = 0.823 and 0.965 and *p* < 0.001). Sensitivity and specificity of miR-33a were 87% and 83% respectively and for miR-122 were 95% and 92% correspondingly for patients (Table 2 and Fig. 2).

**Table 2:** Correlation of miR-33 and miR-122 with biochemical parameters in patients

Variables	miRNA-33		miRNA-122	
	***r***	***p***	***r***	***p***
**BMI (kg/m**^**2**^**)**	0.823	< 0.001	0.965	< 0.001
**FBG (mg/dL)**	0.731	< 0.001	0.863	< 0.001
**HbA1c (%)**	0.885	< 0.001	0.964	< 0.001
**TG (mg/dL)**	0.342	< 0.001	0.291	< 0.001
**cHDL (mg/dL)**	0.149	< 0.001	0.268	< 0.001
**cLDL (mg/dL)**	0.115	< 0.001	0.298	< 0.001

*BMI* body mass index, *FBG* fasting blood glucose, *HbAlc* glycosylated hemoglobin, *TG* triglycerides, *cHDL* high-density lipoprotein cholesterol, *cLDL* low-density lipoprotein, *p p*-value cholesterol, *r* relative correlation coefficient value

## Discussion

Metabolic syndrome (MetS) is a major public health challenge worldwide with an incidence of 20–25% of the world’s population. It is the main cause of obesity, cardiovascular disease, and type 2 diabetes mellitus [19]. The present study comprised 100 Egyptian patients with metabolic syndrome, the majority of patients were females (75% of cases), and the prevalence of obesity was 70%; however, overweight was 30%. These were compatible with numerous studies as determined by Kaur; overweight and obese patients’ cases were 22% and 60% respectively as well as female showed a higher significant difference of obesity more than men [3]. Sliem et al., in 2016, ascertained in their study that women had a higher prevalence of the MetS than men especially in Iran, India, Oman, Pakistan, Saudi, and Egypt; this might be due to cultural barriers to physical activity that have been reported among women. Another study from Turkey reported the highest prevalence of MetS in women (39.6%) than in men (28%) in the Middle East [2]. In Egypt, Nasr et al., in 2010, claimed that the prevalence of obesity was 70.9% among Egyptian patients included in their study and that women had an elevated prevalence of the MetS than men [20]. Moreover, all analyzed biochemical parameters of our subjects showed statistically significant differences compared to controls. In a study that was done by Mohsin et al., in 2007, on 91 participants (95% females) with age < 20 years presented by diabetes type 2, they declared that their anthropometrical and biochemical features of metabolic syndrome were significantly higher in patients than control of *p* value < 0.001 and that HDL levels were higher in females than males; meanwhile, elevated TG levels were reported in 78% of females and 63% of males among the Pakistani population; these criteria were consistent with our anthropometrical and biochemical features which were higher significant in patients than controls of *p* value < 0.001 and HDL levels as well as TG levels were higher in females than males [21]. MicroRNAs (miRNAs) have been recently explored as a regulatory key of gene expression. In the current study, we revealed that miR-33a and miR-122 significantly contributed to the regulation of glucose and lipid metabolism. miR-33a showed downregulation expression in patients compared to controls; meanwhile, miR-122 showed upregulation expression in patients compared to controls within both metabolic disorders. A study done by Rottiers and Näär illustrated that the major metabolic role for miR-33a and miR-122 is the maintenance of cholesterol level and lipid biosynthesis as well as their involvement in insulin signaling and glucose homeostasis in T2DM [22]. Novák et al., in 2014, reviewed in their study the role of both miR-33a and miR-122 in lipid metabolism and reported that the upregulation of miR-33a expression level leads to reduction of fatty acid biosynthesis and in opposition, downregulation of expression level of miR-33a results in an increase of cholesterol level in patients with obesity, yet downregulation of the expression level of miR-122 for many genes, including SREBP, decreases fatty acids and cholesterol synthesis and vice versa its upregulation results in developing obesity [23]. Price et al., in 2018, demonstrated that miR-33a have been shown to control the expression of AMP-activated kinase (*Ampk*α*1*), which is involved in the regulation of lipid metabolism and inhibition of *AMPKα1* by miR-33a may increase intracellular levels of cholesterol and fatty acids. Thus, endogenous inhibition of miR-33a in human hepatic cells increases the degradation of fatty acids and decreases cholesterol level. He also stated that miR-122 plays an important role in regulating serum cholesterol and TG levels by controlling cholesterol biosynthesis leading to very-low-density lipoprotein secretion in the liver and miR-122 inhibition downregulates hepatic expression of several genes involved in the regulation of lipid biosynthesis and reduces cholesterol and lipid accumulation in the liver [24]. As an important biomarker, miRNA-33a plays a remarkable role in insulin signaling and glucose metabolism. Dávalos et al., in 2011, claimed that overexpression of miR-33a reduces insulin signaling in hepatic cell lines, whereas inhibition of endogenous miR-33a enhances glucose pathway [7]. This was more explained by Ramírez et al., in 2013, as he inspected that overexpression of miRNA-33a in human hepatic cells resulted in inhibition of *PCK1* and *G6PC* gene expression, leading to a significant reduction of glucose production [10]. Conversely, downregulation of its expression leads to a significant increase in glucose production and though results in diabetes mellitus. In 2014, Zhang et al. reported in their study the central role of miRNA-33a in regulating glucose synthesis along with activation of the gluconeogenic genes which was negatively regulated by the expression of miR-33a and Srebp2 [6]. On the other hand, miR-122 is significantly correlated to the regulation of glucose metabolism. An essential study was performed by Rashad et al., in 2020, on T2DM Egyptian patients and showed a consistency with our results where miRNA-122 was overexpressed with high significant values in T2DM (*p* < 0.001) compared with the healthy group. Also, clinical, anthropometric, and laboratory characteristics were significantly high of *p* < 0.001 when compared with the control group [25]. Willeit et al. in 2017 study confirmed that upregulation of miRNA-122 is positively associated with glucose metabolism [26]. Numerous studies have been achieved with highly significant differences in different ethnic populations such as Chinese, Caucasian, and Chile when investigating the deregulated overexpression of miRNA-122 with metabolic syndrome parameters related to insulin resistance and lipid profiles [27]. Recently, Lin et al., in 2020, studied the expression level of miRNA-122 in serum samples of 33 participants with their age ranges from 10 to 17 with a mean age of 14.61 ± 2.37 years with obesity. He found that miR-122 was significantly correlated with clinical criteria of obesity (*p* < 0.001) but not with biochemical parameters as triglycerides (TG), high-density lipoprotein cholesterol (HDL-C), and free fatty acids (FFA) [28]. Several clinical perspectives have pointed out that targeting miR-33a and miR-122 may act as promising biomarkers for metabolic disorders including obesity and diabetes mellitus. miRNA profiling could aid for the assessment of the nutritional status and designing a therapeutic strategy suitable for obesity and glucose metabolic diseases. Vasu et al. stated that new approaches integrate RNA sequencing and system biology methodologies will help to elucidate the modulation of gene networks by miRNAs; this may contribute to the regulation of metabolic processes [29].

## Conclusions

This study provides clinical evidence that circulating miRNA-33a and miRNA-122 were remarkably correlated to glucose and lipid metabolism. Alteration in the expression of miRNA-33 or miRNA-122 results in obesity and diabetes. Our findings suggested that further demographic studies should be done to understand more about miRNA-33 and miRNA-122 pathways; this will improve the use of circulating miRNA-33 and miRNA-122 as promising biomarkers of obesity and insulin resistance.

## Data Availability

The data sets generated and/or analyzed during the current study are not publicly available due to patient’s privacy but are available from the corresponding author upon request.

## Abbreviations

*Ampk*α*1*: AMP-activated kinase
*BMI*: Body mass index
Ct: Threshold cycle
FBG: Fasting blood glucose
FFA: Free fatty acids
G6PC: Glucose-6-phosphate
Ht: Height
HDL: High-density lipids
IRS-2: Insulin receptor substrate 2
LDL: Low-density lipids
MetS: Metabolic syndrome
miR-33a: MicroRNA-33a
miR-122: MicroRNA-122
PCK1: Pyruvate carboxy kinase
PK: Pyruvate kinase
RT-PCR: Real-time polymerase chain reaction
Rq: Relative quantification
Sirt6: Sirtuin 6
SD: Standard deviation
*SREBF*: Sterol regulatory element-binding proteins
TC: Total cholesterol
TG: Triglyceride
T2DM: Type 2 diabetes
Wt: Weight
Wc: Wrist circumference
